# Correction to: Long non-coding RNA SLC2A1-AS1 induced by GLI3 promotes aerobic glycolysis and progression in esophageal squamous cell carcinoma by sponging miR-378a-3p to enhance Glut1 expression

**DOI:** 10.1186/s13046-021-02123-1

**Published:** 2021-10-19

**Authors:** Hongtao Liu, Qing Zhang, Yinsen Song, Yibin Hao, Yunxia Cui, Xin Zhang, Xueying Zhang, Yue Qin, Guangzhao Zhu, Feng Wang, Jinghan Dang, Shanshan Ma, Yanting Zhang, Wenna Guo, Shenglei Li, Fangxia Guan, Tianli Fan

**Affiliations:** 1 School of Life Sciences, Zhengzhou University, Zhengzhou, 450001 Henan China; 2 Translational Medicine Research Center, Zhengzhou People’s Hospital, Zhengzhou, 450003 Henan China; 3 Institute of Genomic Medicine, College of Pharmacy, Jinan University, Guangzhou, 510632 Guangdong China; 4 International Cooperative Laboratory of Traditional Chinese Medicine Modernization and Innovative Drug Development of Chinese Ministry of Education (MOE), College of pharmacy, Jinan University, Guangzhou, 510632 Guangdong China; 5 Department of Clinical Medicine, Zhengzhou University, Zhengzhou, 450052 Henan China; 6 Department of Pathology, the First Affiliated Hospital of Zhengzhou University, 40 Daxue Road, Zhengzhou, 450052 Henan China; 7 Department of Pharmacology, School of Basic Medicine, Zhengzhou University, 100 Kexue Road, Zhengzhou, 450001 Henan China

**Correction to: J Exp Clin Cancer Res 40, 287 (2021)**

**https://doi.org/10.1186/s13046-021-02081-8**

Following publication of the original article [1], the authors identified minor errors in the Supplementary Materials section due to mismatch supplementary materials text against the corresponding efiles. The correct texts are as follows:

Additional file 1: Supplementary Tables 1-8

Additional file 2: Supplementary Figures 1-9

The correction does not have any effect on the results or conclusions of the paper. The original article has been corrected.
